# Nicotinamide Riboside and Phycocyanin Oligopeptides Affect Stress Susceptibility in Chronic Corticosterone-Exposed Rats

**DOI:** 10.3390/antiox12101849

**Published:** 2023-10-12

**Authors:** Cemal Orhan, Emre Sahin, Mehmet Tuzcu, Nurhan Sahin, Abdullah Celik, Sara Perez Ojalvo, Sarah Sylla, James R. Komorowski, Kazim Sahin

**Affiliations:** 1 Department of Animal Nutrition, Faculty of Veterinary Medicine, Firat University, Elazig 23119, Turkey; corhan@firat.edu.tr (C.O.); nsahin@firat.edu.tr (N.S.); acelik@beu.edu.tr (A.C.); 2 Department of Animal Nutrition, Faculty of Veterinary Medicine, Bingol University, Bingol 12000, Turkey; esahin@bingol.edu.tr; 3 Department of Biology, Faculty of Science, Firat University, Elazig 23119, Turkey; mtuzcu@firat.edu.tr; 4 Research and Development, Nutrition 21, Harrison, NY 10577, USA; sperezojalvo@nutrition21.com (S.P.O.); ssylla@nutrition21.com (S.S.); jkomorowski@nutrition21.com (J.R.K.)

**Keywords:** corticosterone, phycocyanin, nicotinamide riboside, NAD, hepatic metabolism

## Abstract

Nicotinamide riboside (NR) is an NAD+ precursor capable of regulating mammalian cellular metabolism. Phycocyanin oligopeptide (PC), a phytonutrient found in blue-green algae, has antioxidant and anti-inflammatory properties. This study explored the effects of NR, PC, and their combination on the telomere length as well as inflammatory and antioxidant status of rats under chronic stress conditions (CS). Forty-nine rats were allocated into seven groups: control, chronic stress (CS), CS with NR (26.44 mg/kg), a low dose of 2.64 mg/kg of PC (PC-LD), or a high dose of 26.44 mg/kg PC (PC-HD), NR + PC-LD, and NR + PC-HF. The rats were given daily corticosterone injections (40 mg/kg) to induce stress conditions, or NR and PC were orally administered for 21 days. NR and PC supplementation, particularly NR plus PC, increased the serum antioxidant enzyme activities, hepatic nicotinamide adenine (NAD+) content, and telomere length (*p* < 0.001 for all) compared to the CS group. The levels of serum malondialdehyde (MDA), liver interleukin-6 (IL-6), tumor necrosis factor α (TNF-α), IL-1β, and IL-8 were reduced under the CS condition (*p* < 0.001). In addition, CS decreased the levels of hepatic telomere-related proteins and sirtuins (SIRT1 and 3), whereas administration of NR and PC or their combination to CS-exposed rats increased the levels of telomere-related proteins (e.g., POT1b, TRF1 and TRF2), SIRT3 and NAMPT (*p* < 0.05). In conclusion, NR and PC, especially their combination, can alleviate metabolic abnormalities by enhancing hepatic cytokines, SIRT3, NAMPT, and NAD+ levels in CS-exposed rats. More research is needed to further elucidate the potential health effects of the combination of NR and PC in humans.

## 1. Introduction

Stress is a rapid and coordinated response by an organism to environmental challenges. These responses require the organism to divert energy resources from one tissue to serve the needs of another to counteract the adverse effects of stress. Glucocorticoids (GCs), a class of adrenal steroids released during and after stress, mediate daily physiological changes and coordinate stress. GCs regulate various physiological progressions, mainly by releasing energy stores through gluconeogenesis and suppressing glucose uptake and activity in specific tissues, such as adipose and immune tissue [1,2]. Chronic corticosterone (CORT) exposure increases total body fat mass and decreases insulin sensitivity [3]. The formation of reactive oxygen species (ROS), such as hydrogen peroxide (H_2_O_2_), hydroxyl radicals (HO), and superoxide anion radicals (O^2−^), which lead to lipid peroxidation, is a metabolic consequence of the stress-induced increase in energy production [4]. Additionally, the elevated CORT levels induce a redox imbalance and ROS generation in several tissues [5]. Oxidative stress mediates antioxidant defense system changes, which are also associated with chronic stress [6]. Therefore, the body primarily uses enzymatic and non-enzymatic antioxidant defense systems to neutralize ROS [7]. Cellular oxidative stress may reduce telomere length by attenuating telomerase activity, which controls the de novo synthesis of telomeres [8]. Various age-related diseases have been shown to cause cellular damage [9] and cellular senescence increases due to ROS-induced oxidative stress [10].

Nicotinamide adenine dinucleotide (NAD+) plays a crucial role in energy metabolism and genome integrity by acting as a substrate for different enzyme families, such as sirtuins (SIRTs) and poly(ADP-ribose) polymerases (PARPs) [11]. NAD+ levels decrease in cellular oxidative reactions [12] which may occur during metabolic and age-related diseases and neurodegeneration [11]. SIRTs regulate DNA damage and improve mitochondrial biogenesis [13]. Increased NAD+ levels may improve SIRT activity [12] and prevent telomere shortening under stress conditions [14]. Natural NAD+ precursors such as nicotinamide riboside (NR) can alleviate telomere damage by inhibiting ROS production and mitochondrial impairment [12]. NR supplementation can prevent NAD+ decline in mitochondria and improves mitochondrial activity and biogenesis in hepatocytes, thus ameliorating oxidative-stress-related pathologies and extending one’s health span [15]. Similarly, phycocyanin (PC), a biliprotein derived from spirulina, has potent antioxidant and anti-inflammatory effects [16,17]. PC could activate nuclear transcription factor erythroid-2-like factor 2 (Nrf2)/heme oxygenase-1 (HO-1) signaling, which attenuates oxidative-stress-induced DNA damage in the liver [18]. A recent study by Komorowski et al. [19] indicated that a novel patented PC oligopeptide might be superior to standard PC owing to its high cellular antioxidant capacity. However, the protective effects of the PC oligopeptide and its combination with NR are still unclear. Therefore, this study was conducted to test the effects of PC oligopeptide and NR supplementation on the hepatic abnormalities, NAD+ levels, and underlying mechanisms of rats reared under chronic stress conditions.

## 2. Materials and Methods

### 2.1. Animals

A total of 49 male Wistar albino rats (8 weeks old, 180 ± 20 g) were used, and animals were reared in polypropylene cages conditioned at a temperature of 22 ± 2 °C, with 55 ± 5% humidity and a 12/12 h light/dark cycle. A regular chow diet and water were provided ad libitum. The experiment was conducted under the protocol approved by the Firat University Animal Experiments Ethics Committee (16/03/2020-384972) according to the European Parliament and the Council directive [20] and European Economic Community guidelines [21]. The animal experiments were carried out in the Experimental Research Center of Firat University (FUDAM). All experimental procedures were reported according to Animal Research: Reporting of In Vivo Experiments (ARRIVE) guidelines.

### 2.2. Experimental Design

**Figure 1:**
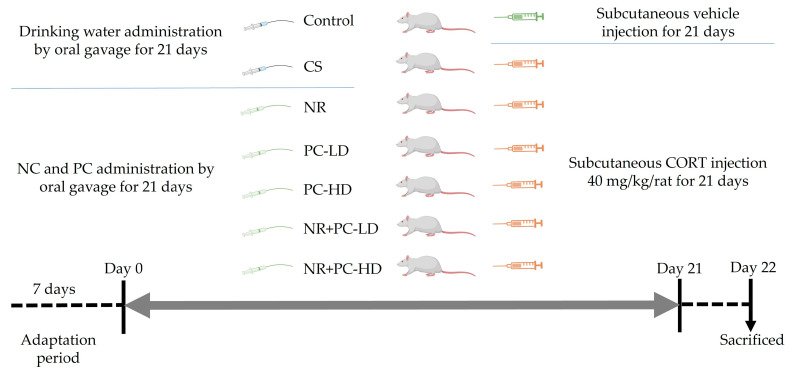
The experimental design. CS: chronic stress induced with corticosterone; NR: nicotinamide riboside (26.44 mg/kg); PC-LD: Phycocyanin oligopeptide, low dose (2.64 mg/kg); PC-HD: Phycocyanin oligopeptide, high dose (26.44 mg/kg). Rats except those of the control group were given daily corticosterone injections (40 mg/kg) to induce stress conditions or NR and PC were orally administered for 21 days.

After a one-week adaptation period, 49 rats were randomly separated into seven groups (*n* = 7) as follows: (1) Control group in whichrats were treated with saline containing tween 80 and DMSO in normal conditions; (2) Chronic stress (CS) group in which rats were injected with corticosterone (CORT, 40 mg/kg) daily for 21 consecutive days; (3) NR group in which rats were injected with CORT and administered NR (26.44 mg/kg); (4) PC-LD group in which rats were injected with CORT and administered a low dose of 2.64 mg/kg of PC; (5) PC-HD group in which rats were injected with CORT and administered a high dose of 26.44 mg/kg PC; (6) NR + PC-LD group in which rats were injected with CORT and administered NR (26.4 mg/kg) and a low dose of 2.64 mg/kg PC; and (7) NR + PC-HD group in which rats were injected with CORT and administered NR (26.44 mg/kg) and a high dose of 26.44 mg/kg PC. The experimental design was schematized in Figure 1. PC and NR were dissolved in drinking water and administered by oral gavage (18-gauge curved metal feeding needle with a bulb-shaped tip) for 21 days (0.5 mL/rat/day). CORT (Sigma–Aldrich Co., St. Louis, MO, USA) was suspended in sterile saline with 0.1% tween 80% and 0.2% DMSO and subcutaneously injected at a volume of 2 mL/kg.

CORT dose was chosen according to Xie et al. [22]. Doses of PC and NR were determined based on the Human Equivalent Dose for Drug Development, according to [23]. Recommended daily human doses (30 or 300 mg HED) were converted to animal doses based on body surface area. A conversion factor of 6.17 was used to convert human doses to rat doses. It was determined that low-dose PC equals 2.64 mg/kg, and NR and high-dose PC equals 26.44 mg/kg for rats.

At the end of the experimental period, on day 22, all animals were sacrificed under xylazine (10 mg/kg)/ketamine hydrochloride (50 mg/kg) anesthesia. Blood samples were collected in biochemical serum tubes, and liver samples were collected. The blood samples were centrifuged, and obtained serum samples were transferred into 1.5 mL tubes. Tissue and serum samples were kept in −80 °C deep freezer for further analysis.

### 2.3. Biochemical Analysis

Serum levels of glucose, cholesterol, blood urea nitrogen (BUN), and creatinine, as well as activities of aspartate aminotransferase (AST) and alanine aminotransferase (ALT) were analyzed with a portable automated chemistry analyzer (Samsung LABGEO PT10, Samsung Electronics Co., Suwon, Republic of Korea) using specific commercial biochemistry profile kits. Repeatability and device/method precision were established according to the commercial kit’s guidelines.

Total serum corticosterone (LifeSpan Biosciences, Seattle, WA, USA) levels and antioxidant enzyme activities [(dismutase (SOD), glutathione peroxidase (GSH-Px), and catalase (CAT)) were measured using the relevant commercial kits (MyBioSource, Inc. San Diego, CA, USA) by enzyme-linked immunosorbent assay (ELISA, Elx-800, Bio-Tek Instruments Inc, Winooski, VT, USA). Additionally, liver NAD+, nicotinamide (NAM), nicotinic acid (NA), and nicotinamide adenine dinucleotide phosphate (NADPH) (MyBioSource, Inc. San Diego, CA, USA) levels were measured by ELISA. All analysis procedures were performed according to the manufacturer’s protocol. The intra- and inter-assay coefficients of variation of CORT (4.2% and 7.5%), SOD (8.0% and 12.0%), GSH-Px (8.0% and 10.0%), CAT (8.0% and 10.0%), NAD (10.0% and 12.0%), NAM (10.0% and 15.0%), NA (4.4% and 5.6%), and NADPH (4.4%, 5.6%) were provided by the manufacturer.

The serum MDA levels were analyzed by high-performance liquid chromatography (HPLC, Shimadzu, Tokyo, Japan) with an SPD-20A UV-visible detector (Shimadzu), C18- DS-3 (5 μm, 4.6 × 250 mm) column, and a column oven (CTO-10ASVP). Tissue samples (300 mg) were mechanically homogenized in a mixture containing 0.5 mL of perchloric acid (0.5 M), 100 µL of 500 μg/mL butylhydroxytoluene, and 2.5 mL of distilled water. Next, the samples were centrifuged (at 4 °C and 2800 g for 10 min), and obtained supernatants were injected (with a volume of 20 µL) into the HPLC system. The mobile phase was 30 mM KH_2_PO_4_-methanol (82.5 + 17.5, *v*/*v*%, pH 3.6), the flow rate was 1.2 mL/min, and detection was at 250 nm.

### 2.4. Relative Telomere Length Measurement

Telomere length in the liver was determined by quantitative real-time PCR (qPCR). Genomic DNA was obtained from the liver tissues of rats using a commercial kit (DNA mini kit, Qiagen, Germany), and the DNA concentration was determined for each sample by a microvolume spectrophotometer (MaestroNano, Maestrogen Inc., USA). For PCR, 20 ng of DNA was diluted in an SYBR Green PCR Kit (QuantiFast, Qiagen, Germany) with AT1 (F: ACGTGTTCTCAGCATCGACCGCTACC, R: AGAATGATAAGGAAAGGGAACAAGAAGCCC, NC_051352.1) and telomere (tel 1 F: GGTTTTTGAGGGTGAGGGTGAGGGTGAGGGTGAGGG; tel 2 R: TCCCGACTATCCCTA TCCCTATCCCTATCCCTATCCCTA, NC_051338.1) primers. For telomere primers, PCR was conducted at 95 °C for 3 min, 95 °C for 15 s (40 cycles), and 54 °C for 1 min on a Rotor-Gene Q machine (Qiagen, Germany). Telomeric DNA was normalized to AT1 quantity. Relative telomere length was expressed as normalized telomere/single gene copy of the AT1 receptor. The relative telomere length of the CORT groups was demonstrated as the relative fold change of the control group.

### 2.5. Western Blotting Analysis

The levels of inflammatory cytokines (interleukin-6 (IL-6), tumor necrosis factor-alpha (TNF-α), IL-1β, and IL-8), telomere shelterin proteins (protection of telomeres protein 1a (POT1a), POTlb, telomeric repeat-binding factor 1 (TRF1), TRF2, and TRF1-interacting nuclear protein 2 (Tin2)), sirtuin-1 (SIRT1), SIRT3, and nicotinamide phosphoribosyltransferase (NAMPT) in the liver were detected by Western blotting. Liver tissues were mechanically homogenized for 30 s (three times for 10 s) in ice-cold radioimmunoprecipitation assay buffer (1:10 *w*/*v*) containing a protease inhibitor cocktail (Calbiochem, San Diego, CA, USA). Next, the samples were agitated for 1 h at 4 °C and centrifuged (15.000× *g* for 20 min at 4 °C). Protein quantification was performed by a microvolume spectrophotometer (MaestroNano, Maestrogen Inc., USA). Then, liver homogenates were mixed with 2× Laemmli buffer (1:1 ratio) and boiled for 5 min in microtubes. Protein samples were separated using 10% sodium dodecylsulfate–polyacrylamide gel electrophoresis (SDS–PAGE) and transferred onto nitrocellulose (NC) membranes. NC membranes were blocked with 5% bovine serum albumin for 2 h. The membranes were incubated with rat-specific primary antibodies diluted to 1:1000 (IL-6 (sc-57315), TNF-α (sc-52746), IL-1β (sc-515598), TRF2 (sc-47693), Tin2 (sc-73177), SIRT3 (sc-365175), NAMPT (sc-393444) (Santa Cruz Biotechnology, Heidelberg, Germany), IL-8 (NBP2-16958), POTlb (NBP2-50255) (Novus Biotech, CO, USA), POT1a (PA5-75366), SIRT1 (MA5-27217), and TRF1 (MA1-46375) (Thermo Fisher Scientific, Waltham, MA, USA)) overnight at 4 °C. After washing, the membranes were incubated with appropriate secondary antibodies diluted to 1:5000 (Santa Cruz Biotechnology) for 2 h. The bands were visualized using a diaminobenzidine substrate solution. Finally, the membranes were scanned, and protein levels were measured densitometrically. Protein loading was checked by using β-actin protein (Santa Cruz Biotechnology, Heidelberg, Germany). The blots were repeated at least three times.

### 2.6. Statistical Analysis

Sample size (N = 49) was calculated based on a power of 85%, an effect size of 0.65, and a *p* value of 0.05 using the G*power program (Version 3.1.9.3) [24]. Given this assumption, a sample size of seven per group was calculated. The data were analyzed using Statistical Analysis Software (IBM SPSS version 22.0). The Shapiro–Wilk and Levene tests were used to determine the normality of the data and homogeneity of variance, respectively. The groups were compared using ANOVA and Tukey post hoc test. *p* < 0.05 was considered to be statistically significant. Data are shown as the mean ± standard deviation.

## 3. Results

### 3.1. The Effects of PC and NR on Body Weight and Biochemical Parameters

CORT administration significantly reduced the rats’ final body weight compared to that of the control group (*p* < 0.001, Table 1). Serum glucose levels were not affected by CORT administration in the CS, NR, PC-LD, and NR + PC-HD groups compared to the control group (*p* > 0.05). However, a dose of 26.44 mg/kg NR plus the administration of 2.64 mg/kg PC decreased the serum glucose level compared to that of the control (*p* < 0.05), CS (*p* < 0.001), and NR groups (*p* < 0.05, Table 1). The serum creatine, BUN, ALT, and AST levels did not differ among the groups (*p* > 0.05).

Changes in the concentrations of serum corticosterone, MDA, SOD, GSH-Px, and CAT to determine the level of stress and antioxidant capacity are shown in Table 2. As expected, the serum corticosterone level was markedly increased in the CORT-treated groups compared to that of the control group (*p* < 0.001). Similarly, the serum MDA levels were elevated after CORT administration compared to those of the control group (*p* < 0.001). The serum MDA levels were reduced by NR and PC supplementation (*p* < 0.001). An apparent decline in the MDA levels was observed in the NR + PC-LD group compared to the NR (*p* < 0.001), PC-LD (*p* < 0.001), and PC-HD (*p* < 0.05) groups. Conversely, the serum SOD, GSH-Px, and CAT levels notably decreased after the CORT injection (*p* < 0.001, Table 2). NR supplementation increased the GSH-Px (*p* < 0.05) and CAT levels (*p* < 0.01), while it did not change the serum SOD levels (*p* > 0.05) compared to the CS group. The PC-PC-LD and PC-HD groups had higher serum SOD and CAT levels than those of the NR group (*p* < 0.05). Furthermore, the combination of NR with each dose of PC prominently boosted the serum SOD and GSH-Px levels compared to the other groups (*p* < 0.05), except for the control group (*p* > 0.05).

### 3.2. The Effects of PC and NR on Liver Nicotinamide Metabolites

We tested the liver NAD+, NAM, NA, NMN, and NADPH levels to assess hepatic NAD+ metabolism. The level of liver NAD+ and its metabolites decreased in the CS group compared to that of the other groups (*p* < 0.05, Table 3). We found that PC supplementation elevated NAD+ and NAM levels in rat liver compared to those under NR supplementation (*p* < 0.05). The NR + PC-LD dose group had markedly higher NAD+ and NADPH levels than the NR (*p* < 0.05) and NR + PC-HD groups (*p* < 0.01). Interestingly, the liver NAM concentrations were similar between the NR and NR + PC-HD groups (*p* > 0.05), while this metabolite was remarkably elevated in the NR + PC-LD group compared to the NR group (*p* < 0.05).

### 3.3. The Effects of PC and NR on Telomere Length

**Figure 2:**
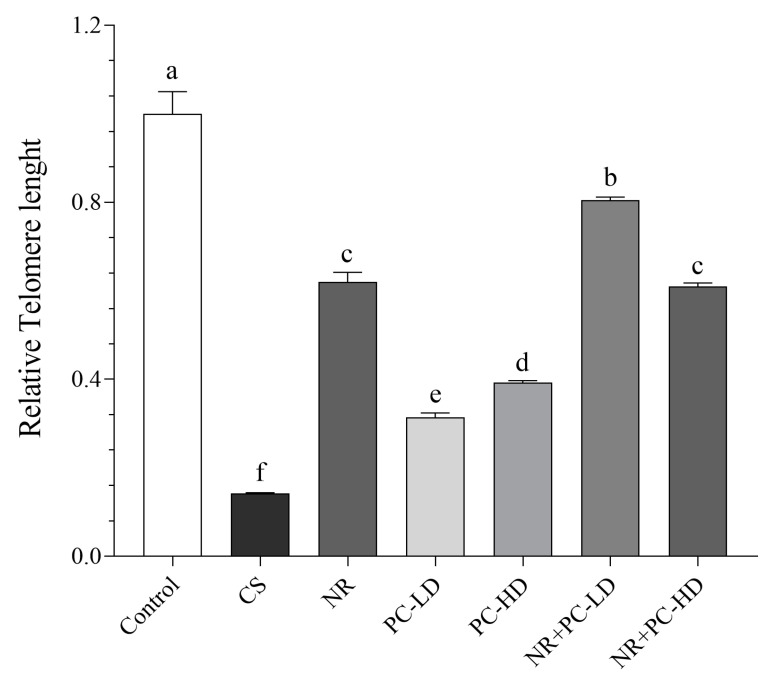
Effects of NR and PC oligopeptides on liver relative telomere length in rats subject to chronic corticosterone (CORT). Data are expressed as a relative fold change compared to the control. The error bars above the lines indicate the standard deviation of the mean. Different symbols (a–f) indicate significant differences among the groups (ANOVA and Tukey post hoc test; *p* < 0.05). CS: chronic stress; NR: Nicotinamide riboside (26.44 mg/kg); PC-LD: Phycocyanin oligopeptide, low dose (2.64 mg/kg); PC-HD: Phycocyanin oligopeptide, high dose (26.44 mg/kg). Rats except those of the control group were given daily corticosterone injections (40 mg/kg) to induce stress conditions, or NR and PC were orally administered for 21 days.

As seen in Figure 2, the CORT injection noticeably decreased the telomere length, and the NR + PC-LD group had the most protective activity against telomere shortening among the CORT-injected groups (*p* < 0.001). The relative telomere length was similar between the NR + PC-HD and NR groups (*p* > 0.05).

### 3.4. The Effects of PC and NR on Inflammatory Mediators

**Figure 3:**
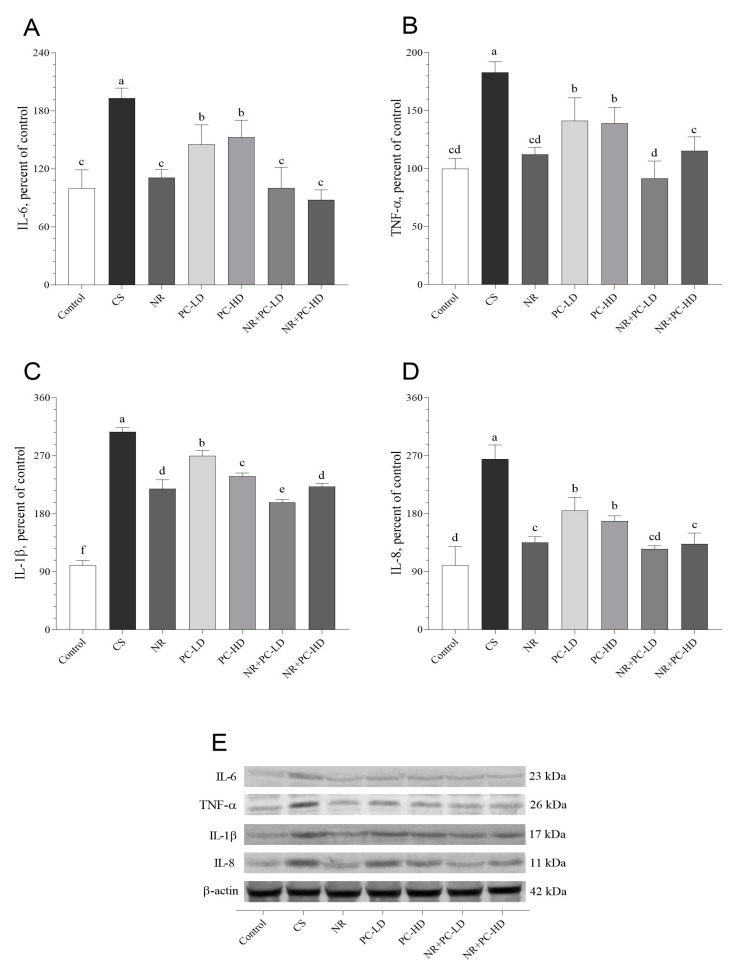
Effects of NR and PC oligopeptides on liver IL-6 (**A**), TNF-α (**B**), IL-1β (**C**), and IL-8 (**D**) levels in rats subject to chronic corticosterone (CORT). The densitometric analysis of the relative intensity according to the control group of the Western blotting bands was performed with β-actin normalization to ensure equal protein loading (**E**). Blots were repeated at least three times (*n* = 3), and a representative blot is shown. Data are expressed as a percent of the control set at 100%. The error bars above the lines indicate the standard deviation of the mean. Different symbols (a–f) indicate significant differences among the groups (ANOVA and Tukey post hoc test; *p* < 0.05). IL-6, interleukin-6; TNF-α, tumor necrosis factor α; IL-1β, interleukin-1β; IL-8, interleukin-8. CS: chronic stress; NR: Nicotinamide riboside (26.44 mg/kg); PC-LD: Phycocyanin oligopeptide, low dose (2.64 mg/kg); PC-HD: Phycocyanin oligopeptide, high dose (26.44 mg/kg). Rats except those of the control group were given daily corticosterone injections (40 mg/kg) to induce stress conditions, or NR and PC were orally administered for 21 days. Full immunoblots presented in Figure S1.

As expected, inflammatory cytokine levels were significantly increased in the CORT-injected rats compared to those of the other groups (Figure 3A–E; *p* < 0.01). The hepatic IL-6 (Figure 3A) levels were similar between the control, NR, NR + PC-LD, and NR + PC-HD groups (*p* > 0.05). NR supplemented with a low dose of PC had an inhibitory effect on the rats’ TNF-α (Figure 3B). It was observed that the NR + PC-LD group had the most inhibitory effect on the IL-1β activity compared to the other CORT-injected groups (*p* < 0.001, Figure 3C). The NR (*p* < 0.01) and NR + PC-HD (*p* < 0.05) groups had higher IL-8 levels than those of the control group (Figure 3D), while NR supplemented with a low dose of PC reduced the hepatic IL-8 level compared to that of the control group (*p* > 0.05).

### 3.5. The Effects of PC and NR on Telomere Sheltering, SIRTs, and NAMPT

**Figure 4:**
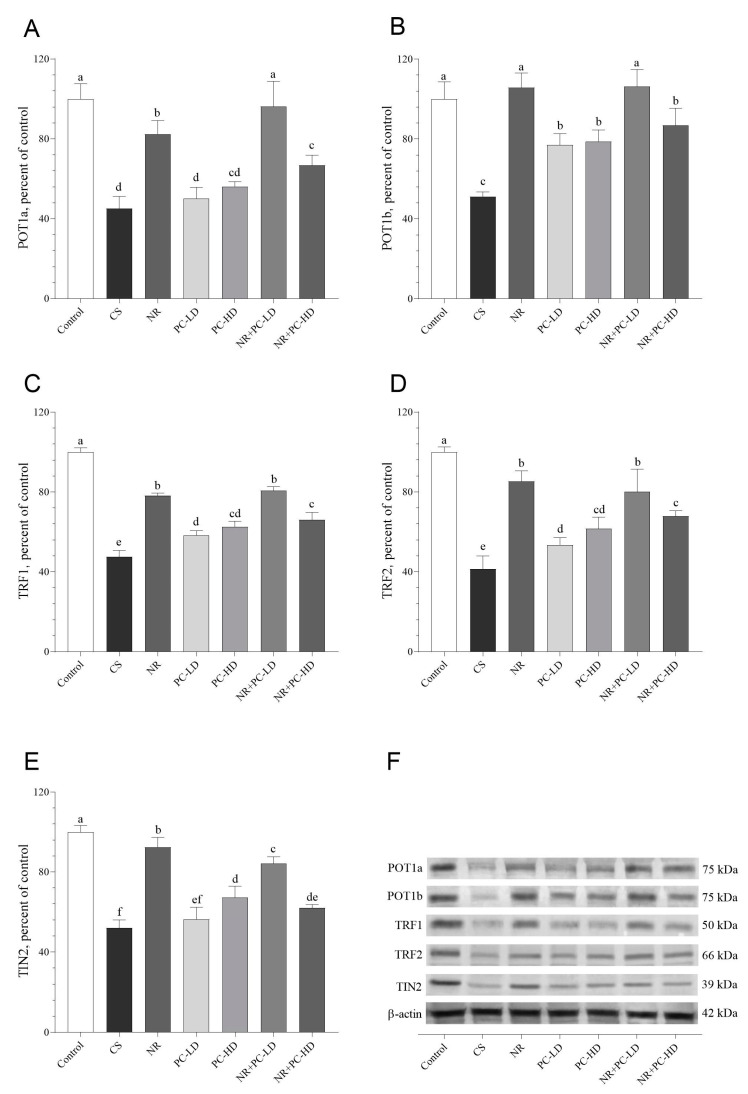
Effects of NR and PC oligopeptides on liver POT1a (**A**), POT1b (**B**), TRF1 (**C**), TRF2 (**D**), and Tin2 (**E**) levels in rats subject to chronic corticosterone (CORT). The densitometric analysis of the relative intensity according to the control group of the Western blotting bands was performed with β-actin normalization to ensure equal protein loading (**F**). Blots were repeated at least three times (*n* = 3), and a representative blot is shown. Data are expressed as a percent of the control set at 100%. The error bars above the lines indicate the standard deviation of the mean. Different symbols (a–f) indicate significant differences among the groups (ANOVA and Tukey post hoc test; *p* < 0.05). POT1a, protection of telomeres protein 1a; POT1b, protection of telomeres protein 1b; TRF1, telomeric repeat-binding factor 1; TRF2, telomeric repeat-binding factor 1; Tin2, TRF1-interacting protein 2. CS: chronic stress; NR: Nicotinamide riboside (26.44 mg/kg); PC-LD: Phycocyanin oligopeptide, low dose (2.64 mg/kg); PC-HD: Phycocyanin oligopeptide, high dose (26.44 mg/kg). Rats except those of the control group were given daily corticosterone injections (40 mg/kg) to induce stress conditions, or NR and PC were orally administered for 21 days. Full immunoblots presented in Figure S2.

**Figure 5:**
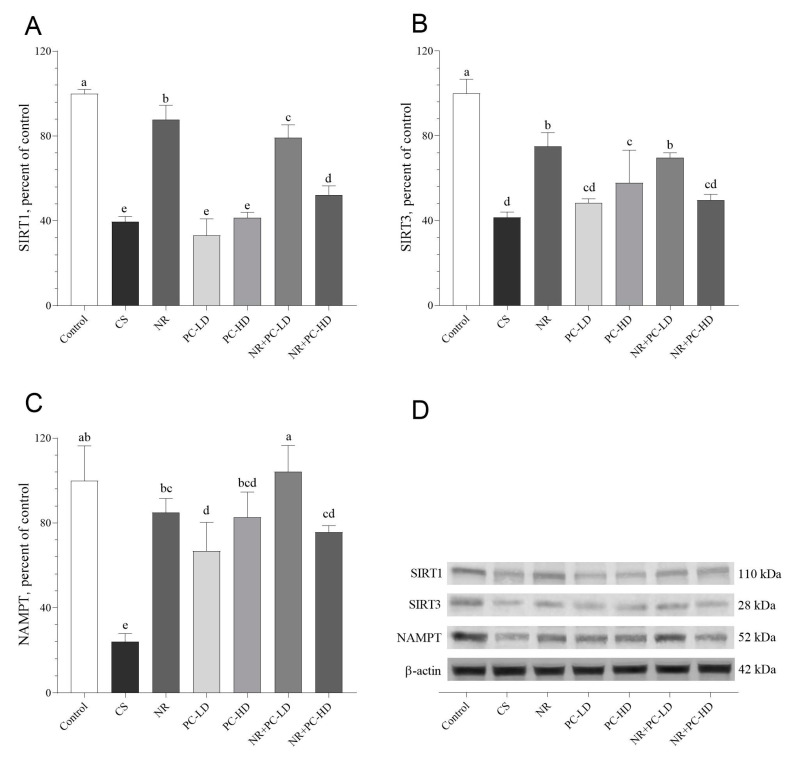
Effects of NR and PC oligopeptides on liver SIRT1 (**A**), SIRT3 (**B**), and NAMPT (**C**) levels in rats subject to chronic corticosterone (CORT). The densitometric analysis of the relative intensity according to the control group of the Western blotting bands was performed with β-actin normalization to ensure equal protein loading (**D**). Blots were repeated at least three times (*n* = 3), and a representative blot is shown. Data are expressed as a percent of the control set at 100%. The error bars above the lines indicate the standard deviation of the mean. Different symbols (a–e) indicate significant differences among the groups (ANOVA and Tukey post hoc test; *p* < 0.05). SIRT1, sirtuin 1; SIRT3, sirtuin 3; NAMPT, nicotinamide phosphoribosyltransferase. CS: chronic stress; NR: Nicotinamide riboside (26.44 mg/kg); PC-LD: Phycocyanin oligopeptide, low dose (2.64 mg/kg); PC-HD: Phycocyanin oligopeptide, high dose (26.44 mg/kg). Rats except those of the control group were given daily corticosterone injections (40 mg/kg) to induce stress conditions, or NR and PC were orally administered for 21 days. Full immunoblots presented in Figure S3.

We next analyzed the protein levels of the telomere shelterin complexes by Western blotting. As shown in Figure 4 and Figure 5, NR or NR + PC supplementation increased the telomere shelterin complex, SIRT1, SIRT3, and NAMPT protein levels, which were reduced after the administration of CORT (*p* < 0.05). The control and NR + PC-LD groups had similar POT1a protein levels (*p* > 0.05, Figure 4A). Additionally, we detected that the POT1b levels did not differ between the control, NR, and NR + PC-LD groups (*p* > 0.05, Figure 4B). Although the NR and NR + PC-LD groups had significantly higher TRF1 (Figure 4C) and TRF2 (Figure 4D) levels than the other CORT-administered groups (*p* < 0.01 for all), these groups could not reach the TRF1 and TRF2 levels of the control group. Moreover, we found that Tin2 levels in the NR + PC-LD group noticeably increased compared to those in the CS group (*p* < 0.05, Figure 4E). Unfortunately, none of the CORT-injected groups had significantly elevated Tin2, TRF1, or TRF2 levels compared to those of the control (*p* < 0.05).

In order to explore the effect of NR and PC oligopeptides on telomere lengthening, we measured the levels of SIRT1, SIRT3, and NAMPT, which are NAD+-related enzymes. The NR group had the highest SIRT1 levels among the CORT-injected groups (Figure 5A). Any doses of PC without NR did not alter the SIRT1 level compared to the CS group (*p* > 0.05, Figure 5A). Similarly, the PC-LD and NR + PC-HD groups did not significantly increase the SIRT3 levels compared to the CS group (*p* > 0.05, Figure 5B). However, the NR and NR + PC-LD groups had the highest SIRT3 levels among the CORT-injected groups (*p* < 0.05, Figure 5B). On the other hand, the NR + PC-LD group remarkably boosted the NAMPT levels compared to the other groups, except for that of the control (*p* < 0.05, Figure 5C). Additionally, the NR + PC-LD group had a higher NAMPT level than the control group, but the difference was not statistically significant (*p* > 0.05, Figure 5C).

## 4. Discussion

This study showed that chronic CORT exposure increased oxidative stress by increasing the MDA content and decreasing the antioxidant activity of SOD, CAT, and GSH-Px. At the same time, it also increased serum corticosterone concentrations and liver anti-inflammatory (TGF-β) and pro-inflammatory (TNF-α, IL-1β, IL-6) cytokines levels. Similar to our results, it has been well documented in previous studies that chronic CORT exposure increases MDA levels [25], stimulates inflammatory cytokines [25,26], suppresses antioxidant enzyme activity [26], and accelerates telomere shortening [22,26]. As in our findings, it has been reported that NR and PC may exert inhibitory effects on oxidative-stress-induced tissue damage in animals [16,27] and humans [12,27] by attenuating short-telomere-induced DNA damage [12].

CORT-induced oxidative and inflammatory status probably increases PARP1 and cyclic ADP ribose hydrolase (CD38) activation, which are the primary consumers of NAD+, and restrict NAD+ bioavailability to SIRTs [28] that prevent liver damage, fat accumulation, and fibrosis [29]. Therefore, in the present study, the limited NAD+ bioavailability caused by CORT-induced liver inflammation may have reduced the SIRT1 and SIRT3 levels in the liver of CORT rats. NR can enter the NAD+ salvage pathway via phosphorylation to NMN by nicotinamide riboside kinases (NRKs), and NMN is converted into NAD+ to feed the NAD+ biosynthesis cycle [30,31]. Oral NR supplementation achieves the replenishment of cellular NAD+ reduced by inflammation and may diminish circulating inflammatory cytokine activities [32]. Thus, NR can attenuate oxidative inflammatory tissue damage by inhibiting mitochondrial ROS production and reducing MDA levels while elevating SOD levels [33,34]. Analogously, PC supplementation boosts antioxidant enzyme levels in the liver and alleviates hepatic oxidative stress due to its radical scavenging, lipid peroxidation prevention [35], and mitochondrial respiratory complex protective activity [36]. The anti-inflammatory ability of PC may stem from its inhibitory action on activating programmed cell death five protein (PCDP5)/nuclear factor κ-B or tool-like receptor 2/NF-κ-B pathways [37,38].

In the present study, NR plus PC supplementation effectively increased liver NAD+, NAM, and NADPH levels. Similar to our results, it has been shown previously that PC shows inhibitory activity on NADPH oxidase (NOX) [39], producing ROS by utilizing NADPH [28]. Fan et al. [40] showed that NOX inhibition augments NADPH and NAD+ levels. Based on Fan et al. [40]’s report, we assumed that the NADPH levels increased following NOX inhibition by PC, and the NAD+ and NAM levels were elevated in parallel depending on the reduced NADPH requirement. The elevated NAM level might have stimulated NAMPT upregulation in the group with NR supplemented with a low dose of PC to promote the NAD+ salvage pathway. NAMPT can mediate the activity of NAD+-dependent enzymes and alleviates hepatic steatosis in a SIRT-dependent manner in mice [41]. NAMPT overexpression can independently influence liver regeneration, and this regeneration process may also be restored by NR treatment in NAMPT knockout mice through SIRT1-dependent pathways [42]. In contrast to other spirulina-derived PCs [43], we found that novel PC oligopeptides could not have effectively stimulated SIRT1 while increasing the SIRT3 levels in the liver. Moreover, NR+PC supplementation was not as effective as NR supplementation in elevating SIRT3 levels. Under inflammatory conditions, increased NAM levels by phytochemical administration may negatively affect SIRT1 activity due to the activation of the PARP1-dependent anti-inflammatory response pathway [44]. Although we could not demonstrate why SIRT1 was not stimulated by PC, our results suggested that the PC oligopeptide exerts SIRT1-independent anti-inflammatory effects in the liver of CORT-treated rats.

We also explored the effects of NR and PC on telomere shelterin complex proteins. Recently, Shen et al. [45] showed that CORT exposure impaired the telomere shelterin protein complex and reduced the telomere length in the liver of rats. TRF1 has both lengthening and shortening effects on telomeres to avoid DNA damage and protect telomere stability [46]. Similarly to our results, Badmus et al. [47] reported that liver TRF1 levels decreased after four weeks of CORT feeding in broiler chickens. TRF2 positively regulates telomere function and continually interacts with TRF1 and POT1 to control telomere length [48]. An in vitro study showed that oxidative damage could interfere with telomere length by suppressing TRF2 activity [49]. Xie et al. [22] indicated that CORT exposure reduced liver TRF2 activity in rats. In parallel with these results, we found that the POT1a and POT1b levels were reduced after our CORT injection because POT1 binding to the TRF1 and TRF2 complex is mediated by TIN2-interacting protein (TPP1) and TIN2 [50]. The interaction between POT1, TPP1, and TIN2 orchestrates the functions of telomere shelterin complex proteins and telomere integrity [51]. Shen et al. [45] demonstrated that liver TIN2 and POT1a levels decreased after CORT exposure in rats, and this report supports our findings. Promoting NAD+ metabolism after NR supplementation may prevent ROS-induced DNA damage and mitochondrial impairment in DC fibroblasts [12]. Because NR activates the NAD+/SIRT1 pathway, it increases mitochondrial biogenesis and decreases ROS generation; thus, NR may prevent telomere shortening [12]. On the other hand, PC supplementation presumably regulates telomere shelterin proteins, owing to its potent antioxidant activity [19,52] and protective effect on the mitochondrial respiratory complex [36] rather than the SIRT1-dependent pathway. Although we did not measure the liver mTOR levels, PC might have increased the telomere length by diminishing mTOR complex activation, as reported previously by Joly et al. [53]. Furthermore, we elucidate that PC partly stimulated SIRT3 activity, which improved mitochondrial biogenesis and antioxidant defense [54]. Collectively, NR plus PC administration may have concurrently prevented hepatic telomere shortening in CORT rats.

Interestingly, combining NR with a high-dose PC oligopeptide was less effective than NR with a low-dose PC oligopeptide. This is probably because antioxidants exert hormetic effects on the redox state, and their mechanisms of action may vary depending on the dose [55]. A previous study by Kourtzidis et al. [56] reported that high-dose NR supplementation may cause a hormetic impact that results in a non-optimal redox state in the liver of rats. Despite the low possibility of PC exhibiting a hormetic effect [17], in the current study, it is possible that combined supplementation of NR and high-dose PC could have initiated the induction of a hormetic pathway depending on the antioxidant overload. Thus, the protective effect of NR on hepatic telomere length, inflammation, and NAD+ metabolism may have been attenuated due to its insufficient capability to protect the redox balance of NR supplemented with a high dose of PC.

## 5. Conclusions

In conclusion, the present study showed that NR and PC oligopeptide supplementation in CORT-treated rats might be relevant to regulating hepatic telomere shortening and energy metabolism by modulating the response of the liver through a metabolic increase in both the cellular NAD+ availability and hepatic cytokines, SIRT-3, and NAMPT pathways. However, more preclinical and clinical studies are needed to reveal the molecular mechanisms of action of these combinations.

## Figures and Tables

**Table 1:** Effects of nicotinamide riboside and phycocyanin oligopeptides on serum biochemical parameters in rats subject to chronic corticosterone.

	Groups						
	Control	CS	NR	PC-LD	PC-HD	NR + PC-LD	NR + PC-HD
Final BW, g	276.57 ± 9.85 ^a^	210.43 ± 8.28 ^b^	222.86 ± 23.11 ^b^	221.29 ± 11.25 ^b^	221.86 ± 9.92 ^b^	225.14 ± 13.23 ^b^	217.86 ± 19.42 ^b^
Glucose, mg/dL	119.14 ± 8.43 ^ab^	127.86 ± 5.05 ^a^	119.86 ± 7.10 ^ab^	118.57 ± 2.64 ^abc^	114.14 ± 5.24 ^bc^	109.29 ± 5.47 ^c^	118.29 ± 4.68 ^bc^
Creatine, mg/dL	0.40 ± 0.06	0.38 ± 0.07	0.37 ± 0.08	0.40 ± 0.08	0.39 ± 0.06	0.36 ± 0.09	0.45 ± 0.05
BUN, mg/dL	21.46 ± 3.57	23.01 ± 2.32	22.90 ± 1.87	22.13 ± 2.52	21.59 ± 3.00	22.20 ± 4.11	23.17 ± 3.65
ALT, U/L	95.57 ± 8.42	98.14 ± 12.81	98.00 ± 5.55	98.71 ± 6.82	98.03 ± 7.36	96.10 ± 5.78	98.72 ± 4.79
AST, U/L	110.69 ± 11.79	117.46 ± 7.53	114.71 ± 10.92	112.60 ± 13.13	117.43 ± 15.25	114.06 ± 12.59	116.20 ± 19.06

Data are presented as the mean and standard deviation. ^a–c^: Means in the same line without a common superscript differ significantly (*p* < 0.05, ANOVA and Tukey post hoc test). CS: chronic stress induced with corticosterone; NR: Nicotinamide riboside (26.44 mg/kg); PC-LD: Phycocyanin oligopeptide, low dose (2.64 mg/kg); PC-HD: Phycocyanin oligopeptide, high dose (26.44 mg/kg). Rats except those of the control group were given daily corticosterone injections (40 mg/kg) to induce stress conditions, or NR and PC were orally administered for 21 days. BUN: Blood urea nitrogen; ALT: Alanine transaminase; AST: Aspartate aminotransferase.

**Table 2:** Effects of nicotinamide riboside and phycocyanin oligopeptides on serum biochemical parameters in rats subject to chronic corticosterone.

	Groups						
	Control	CS	NR	PC-LD	PC-HD	NR + PC-LD	NR + PC-HD
Corticosterone, ng/mL	46.33 ± 5.82 ^b^	110.22 ± 6.15 ^a^	102.16 ± 8.68 ^a^	104.48 ± 3.67 ^a^	105.91 ± 6.39 ^a^	105.46 ± 8.13 ^a^	104.99 ± 5.34 ^a^
MDA, nmol/mL	0.55 ± 0.07 ^f^	1.97 ± 0.13 ^a^	1.74 ± 0.07 ^b^	1.53 ± 0.07 ^c^	1.41 ± 0.07 ^cd^	1.26 ± 0.07 ^e^	1.28 ± 0.09 ^de^
SOD, U/mL	128.00 ± 5.48 ^a^	54.54 ± 6.39 ^e^	61.36 ± 4.5 ^e^	72.70 ± 3.05 ^d^	86.41 ± 6.34 ^c^	96.84 ± 6.77 ^b^	98.27 ± 4.65 ^b^
GSH-Px, U/mL	64.87 ± 3.08 ^a^	19.62 ± 1.37 ^e^	26.28 ± 1.98 ^d^	31.76 ± 4.96 ^cd^	37.10 ± 2.80 ^c^	44.68 ± 5.58 ^b^	45.98 ± 4.10 ^b^
CAT, U/mL	164.98 ± 6.13 ^a^	102.03 ± 4.80 ^e^	114.69 ± 4.82 ^d^	124.55 ± 5.48 ^c^	131.43 ± 3.76 ^bc^	140.55 ± 6.76 ^b^	139.05 ± 7.81 ^b^

Data are presented as the mean and standard deviation. ^a–f^: Means in the same line without a common superscript differ significantly (*p* < 0.05, ANOVA and Tukey post hoc test). CS: chronic stress induced with corticosterone; NR: Nicotinamide riboside (26.44 mg/kg); PC-LD: Phycocyanin oligopeptide, low dose (2.64 mg/kg); PC-HD: Phycocyanin oligopeptide, high dose (26.44 mg/kg). Rats except those of the control group were given daily corticosterone injections (40 mg/kg) to induce stress conditions or NR and PC were orally administered for 21 days. MDA: Malondialdehyde; SOD: Superoxide dismutase; GSH-Px: Glutathione peroxidase; CAT: Catalase.

**Table 3:** Effects of nicotinamide riboside and phycocyanin oligopeptides on serum biochemical parameter in rats subject to chronic corticosterone.

	Groups						
	Control	CS	NR	PC-LD	PC-HD	NR + PC-LD	NR + PC-HD
NAD^+^, µmol/g	0.76 ± 0.05 ^a^	0.33 ± 0.04 ^f^	0.59 ± 0.04 ^c^	0.42 ± 0.05 ^e^	0.49 ± 0.06 ^de^	0.67 ± 0.05 ^b^	0.55 ± 0.06 ^cd^
NAM, µmol/g	2.36 ± 0.15 ^a^	1.01 ± 0.08 ^e^	1.60 ± 0.06 ^c^	1.31 ± 0.09 ^d^	1.43 ± 0.13 ^d^	1.78 ± 0.10 ^b^	1.66 ± 0.10 ^cb^
NA, µmol/g	1.79 ± 0.09 ^a^	1.22 ± 0.12 ^d^	1.63 ± 0.09 ^abc^	1.46 ± 0.10 ^c^	1.57 ± 0.17 ^bc^	1.71 ± 0.13 ^ab^	1.73 ± 0.12 ^ab^
NMN, µmol/g	0.45 ± 0.05 ^a^	0.15 ± 0.02 ^e^	0.30 ± 0.02 ^bc^	0.20 ± 0.02 ^de^	0.24 ± 0.03 ^cd^	0.36 ± 0.05 ^b^	0.32 ± 0.07 ^b^
NADPH, nmol/g	62.65 ± 4.13 ^a^	30.84 ± 3.52 ^e^	44.42 ± 2.31 ^c^	39.88 ± 3.04 ^cd^	41.38 ± 3.57 ^cd^	52.52 ± 5.39 ^b^	37.89 ± 2.23 ^d^

Data are presented as the mean and standard deviation. ^a–f^: Means in the same line without a common superscript differ significantly (*p* < 0.05, ANOVA and Tukey post hoc test). CS: chronic stress induced with corticosterone; NR: Nicotinamide riboside (26.44 mg/kg); PC-LD: Phycocyanin oligopeptide, low dose (2.64 mg/kg); PC-HD: Phycocyanin oligopeptide, high dose (26.44 mg/kg). Rats except those of the control group were given daily corticosterone injections (40 mg/kg) to induce stress conditions or NR and PC were orally administered for 21 days. Nicotinamide adenine dinucleotide; NAD+: Nicotinamide adenine dinucleotide; NAM: Nicotinamide; NA: Nicotinic acid; NMN: Nicotinamide mononucleotide; NADPH: nicotinamide adenine dinucleotide phosphate.

## Data Availability

The datasets for this study can be found in the article.

## Supplementary Materials

The following supporting information can be downloaded at: https://www.mdpi.com/article/10.3390/antiox12101849/s1, Figure S1: Full immunoblots related to Figure 2 [liver IL-6 (A), TNF-α (B), IL-1β (C), IL-8 (D) and (E) β-actin levels]. The densitometric analysis of the relative intensity according to the control group of the western blot bands was performed with β-actin normalization to ensure equal protein loading. How many kDa are shown in each blot’s representative. IL-6, interleukin-6; TNF- α, tumor necrosis factor α; IL-1β, interleukin-1β; IL-8, interleukin8.; Figure S2: Full immunoblots related to Figure 3 [liver POT1a (A), POT1b (B), TRF1 (C), TRF2 (D) TIN2 (E) and β-actin (F) levels]. The densitometric analysis of the relative intensity according to the control group of the western blot bands was performed with β-actin normalization to ensure equal protein loading. How many kDa are shown in each blot’s representative. POT1a, protection of telomeres protein 1a; POT1b, protection of telomeres protein 1b; TRF1, telomeric repeat-binding factor 1; TRF2, telomeric repeat-binding factor 1; TIN2, TRF1- interacting protein 2.; Figure S3: Full immunoblots related to Figure 4 [liver SIRT1 (A), SIRT3 (B), NAMPT (C) and β-actin (D) levels]. The densitometric analysis of the relative intensity according to the control group of the western blot bands was performed with β-actin normalization to ensure equal protein loading. How many kDa are shown in each blot’s representative. SIRT1, sirtuin 1; SIRT3, sirtuin 3; NAMPT, Nicotinamide phosphoribosyltransferase.
