# Supplementary material for: Nicotinamide Riboside and Phycocyanin Oligopeptides Affect Stress Susceptibility in Chronic Corticosterone-Exposed Rats
Source: Antioxidants (Basel). 2023 Oct 12;12(10):1849. doi: 10.3390/antiox12101849 (PMC10604757; doi:10.3390/antiox12101849)
Supplement: Supplementary file 1 [file antioxidants-12-01849-s001.zip › antioxidants-2643547-supplementary.pdf]

# Nicotinamide riboside and phycocyanin oligopeptides affect stress susceptibility in chronic corticosterone-exposed rats

**Cemal Orhan[1], Emre Sahin[2], Mehmet Tuzcu[3], Nurhan Sahin[1], Abdullah Celik[1], Sara Perez Ojalvo[4], Sarah Sylla[4], James R. Komorowski[4] and Kazim Sahin[1,*]**

[1]Department of Animal Nutrition, Faculty of Veterinary Medicine, Firat University, Elazig, Turkey

[2]Department of Animal Nutrition, Faculty of Veterinary Medicine, Bingol University, Bingol, Turkey

[3]Department of Biology, Faculty of Science, Firat University, Elazig, Turkey

[4]Research and Development, Nutrition 21, Harrison, NY 10577, USA

*****Corresponding author:

Kazim Sahin, DVM, Ph.D., F.A.C.N. Member of The Turkish Academy of Sciences,

Professor of Nutrition Veterinary Faculty Firat University 23119 Elazig, Turkey. Phone: +90-532-7473506, Phone: +904242370000/3938

Email: nsahinkm@yahoo.com, ksahin@firat.edu.tr

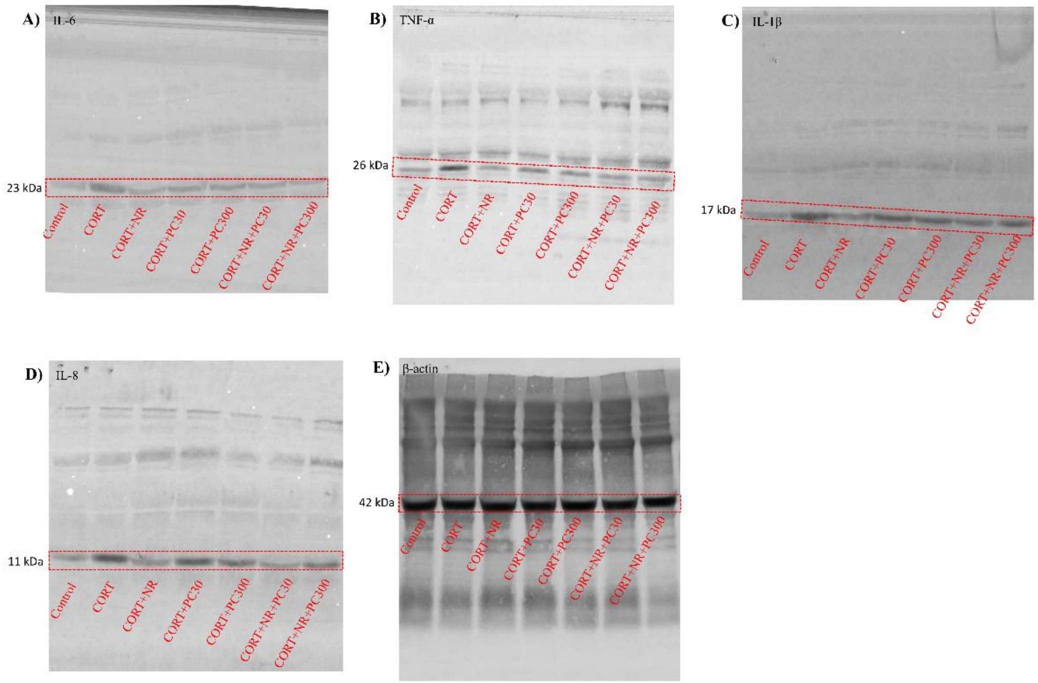


**Figure S1.** Full immunoblots related to Figure 2 [liver IL-6 (A), TNF-α (B), IL-1β (C), IL-8 (D) and (E) β-actin levels]. The densitometric analysis of the relative intensity according to the control group of the western blot bands was performed with β-actin normalization to ensure equal protein loading. How many kDa are shown in each blot's representative. IL-6, interleukin-6; TNF- α, tumor necrosis factor α; IL-1β, interleukin-1β; IL-8, interleukin-8.

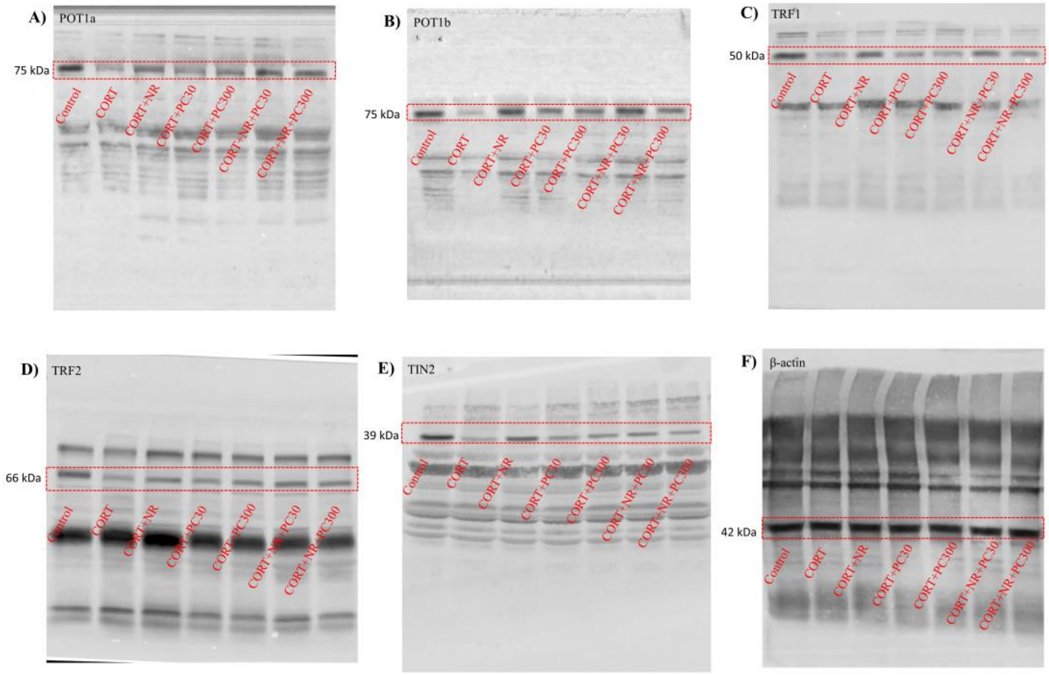


**Figure S2.** Full immunoblots related to Figure 3 [liver POT1a (A), POT1b (B), TRF1 (C), TRF2 (D) TIN2 (E) and β-actin (F) levels]. The densitometric analysis of the relative intensity according to the control group of the western blot bands was performed with β-actin normalization to ensure equal protein loading. How many kDa are shown in each blot's representative. POT1a, protection of telomeres protein 1a; POT1b, protection of telomeres protein 1b; TRF1, telomeric repeat-binding factor 1; TRF2, telomeric repeat-binding factor 1; TIN2, TRF1-interacting protein 2.

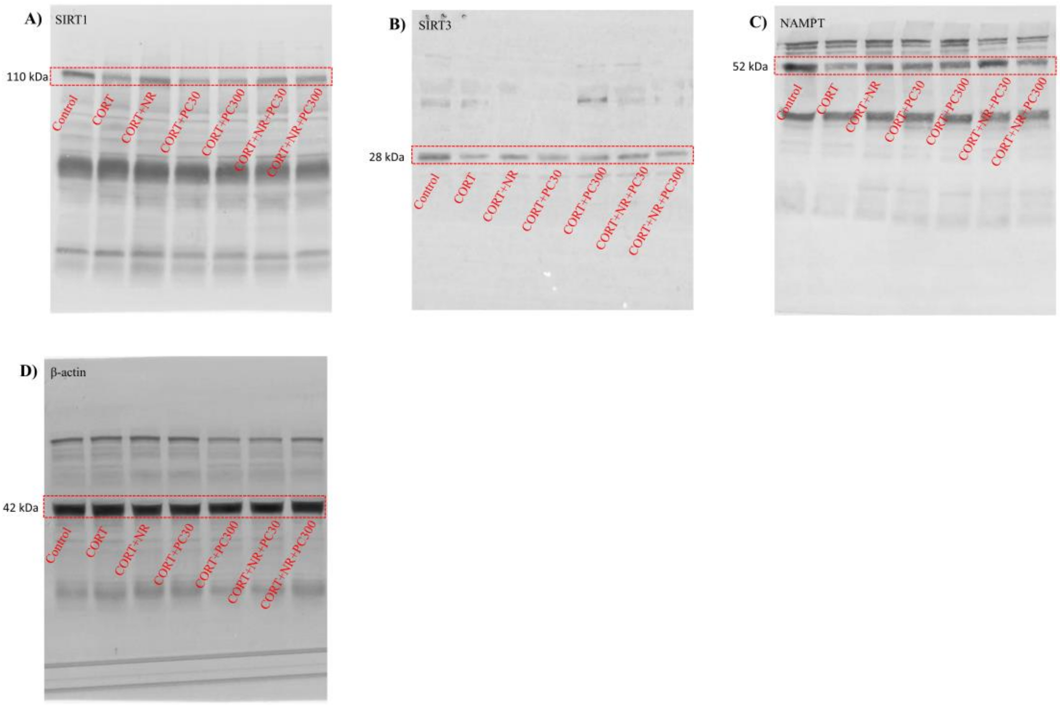


**Figure S3.** Full immunoblots related to Figure 4 [liver SIRT1 (A), SIRT3 (B), NAMPT (C) and β-actin (D) levels]. The densitometric analysis of the relative intensity according to the control group of the western blot bands was performed with β-actin normalization to ensure equal protein loading. How many kDa are shown in each blot's representative. SIRT1, sirtuin 1; SIRT3, sirtuin 3; NAMPT, Nicotinamide phosphoribosyltransferase.
